# TNF-α blockade with adalimumab restores bone metabolism in rheumatoid arthritis by regulating cytokines and inflammatory cell infiltration

**DOI:** 10.3389/fimmu.2026.1866519

**Published:** 2026-07-23

**Authors:** Akilan Krishnamurthy, Shankar Revu, Petra Neregård, Aase Hensvold, Erik af Klint, Dimitrios Makrygiannakis, Anca I. Catrina, Ioannis Parodis

**Affiliations:** 1 Division of Rheumatology, Department of Medicine Solna, Karolinska Institutet, Karolinska University Hospital, and Center for Molecular Medicine (CMM), Stockholm, Sweden; 2 Department of Rheumatology, Faculty of Medicine and Health, Örebro University, Örebro, Sweden

**Keywords:** anti-TNF-α, biologics, bone erosion, osteoblasts, osteoclasts, rheumatoid arthritis, synovial inflammation

## Abstract

**Objective:**

To investigate the mechanisms underlying the bone-sparing effects of the anti-TNF antibody adalimumab in rheumatoid arthritis (RA) by examining synovial tissue and its direct effect on osteoblasts and osteoclasts.

**Methods:**

Paired synovial biopsies from 12 patients with RA were obtained before and 8 weeks after initiation of adalimumab. Immune-cell infiltrates (CD163^+^ macrophages, CD3^+^ lymphocytes) and expression of osteoprotegerin (OPG), receptor activator of nuclear factor κB (RANK), and its ligand (RANKL) were assessed by immunohistochemistry. *In vitro*, the effect of adalimumab on RANKL and OPG expression in SaOS-2 cells (± TNF-α) was evaluated by western blotting. Its effect on osteoclastogenesis and bone resorption were examined using CD14^+^ monocyte-derived macrophages cultured with RANKL and M-CSF.

**Results:**

Adalimumab treatment led to a marked clinical improvement, with median DAS28 decreasing from 5.4 to 3.1. Semiquantitative analysis of synovial biopsies revealed a significant decrease in CD163^+^ macrophages, while CD3^+^ T-cells remained unchanged. The RANKL/OPG ratio decreased significantly, which was accompanied by increased OPG expression in synovial tissue. *In vitro*, adalimumab significantly decreased RANKL expression and the RANKL: OPG ratio in TNF-α primed SaOS-2 cells, while also modulating pro-osteoclastogenic cytokines. In osteoclast assays, adalimumab inhibited macrophage-derived osteoclast differentiation and suppressed bone resorption under both low and high RANKL conditions.

**Conclusion:**

Adalimumab decreased synovial macrophage infiltration while it modulates pro-osteoclastogenic cytokines and inhibits osteoclastogenesis. These combined effects explain how TNF-α blockade ameliorates joint inflammation and prevents structural damage.

## 1 Introduction

Rheumatoid arthritis (RA) is a systemic autoimmune disease characterized by persistent synovial inflammation that leads to cartilage destruction and progressive bone erosion (1). Anti-citrullinated protein antibodies (ACPAs) are a major risk factor for erosive disease (2). ACPAs and other RA-associated autoantibodies may contribute to inflammation and structural damage by promoting increase in osteoclast differentiation and bone-resorption through tumor necrosis factor-alpha (TNF-α), interleukin (IL)-8, and lipid mediated pathways (3–8).

TNF-α is a central cytokine in RA pathogenesis, driving synovial hyperplasia, joint inflammation, and osteoclast activation (9, 10). One of its major effects is disruption of the balance between receptor activator of nuclear factor κB ligand (RANKL) and osteoprotegerin (OPG). RANKL promotes osteoclast differentiation by binding the RANK receptor on osteoclast precursors, whereas OPG, a soluble decoy receptor, inhibits this process by binding to RANKL (11–15).

Synovial macrophages represent key effector cells within the TNF-driven inflammatory microenvironment of RA, and single-cell RNA sequencing has indicated that CD206^+^CD163^+^ macrophages are significantly enriched in RA synovial tissue (16). They perpetuate inflammation through the production of TNF-α, IL-6, and IL-8, and orchestrate the activation states of fibroblasts, lymphocytes, and osteoclast precursors (16, 17). Their abundance in the synovial tissue correlates strongly with increased disease activity (16, 18). Importantly, synovial tissue macrophages belong to the monocyte–macrophage lineage and can differentiate into functional osteoclasts in the presence of RANKL and macrophage colony stimulating factor (M-CSF), directly linking synovial inflammation to pathological bone resorption (19, 20).

It is well established that the RANKL/RANK/OPG axis plays a pivotal role in bone loss in RA (15, 21). Osteoblasts and osteocytes, the bone forming cells are major sources of both RANKL and OPG, and inflammatory cytokines such as TNF-α shift osteoblasts towards a pro-osteoclastogenic phenotype by increasing RANKL expression and reducing OPG production (21, 22). In the inflamed synovium, fibroblasts, vascular endothelial cells and activated T and B lymphocytes further contribute to RANKL production, collectively promoting osteoclast differentiation and bone erosion. Bone loss can occur through TNF-α-dependent and independent pathways, indicating that inflammation and bone destruction may be partially uncoupled; notably, TNF-blocking therapy can inhibit bone erosion even in the absence of marked clinical improvement (15, 23–25).

Adalimumab, a fully human monoclonal antibody targeting TNF-α, has demonstrated robust efficacy in reducing disease activity and slowing radiographic progression in RA (26, 27). However, the mechanisms by which TNF-α inhibition translates into structural protection remain incompletely understood. Synovial tissue analysis offers a unique opportunity to elucidate these mechanisms, as changes in cellular composition and molecular pathways within the joint may reveal how TNF-α blockade modulates bone-related processes (28).

In this study, we aimed (i) to characterize synovial tissue changes in patients with RA treated with adalimumab, focusing on immune-cell infiltration and expression of bone-related markers, and (ii) to investigate the direct effects of adalimumab on the RANKL/RANK/OPG axis and osteoclast differentiation *in vitro*. By integrating tissue-level observations with functional assays, we sought to clarify the pathways through which TNF inhibition mitigates bone erosion, thereby informing strategies to optimize structural outcomes in RA.

## 2 Materials and methods

### 2.1 Patients

Twelve patients with RA (5 men and 7 women; median disease duration: 5 years) who had failed treatment with at least one synthetic disease-modifying antirheumatic drug (DMARD), either as monotherapy or in combination with a biologic other than adalimumab, were enrolled. All patients received adalimumab 40 mg subcutaneously every other week. Concomitant synthetic DMARD therapy was maintained at a stable dose for at least one month before initiation of adalimumab and throughout the study. Prednisolone (≤10 mg/day) and intra-articular corticosteroid injections in joints other than the biopsied joint were permitted as clinically indicated.

Synovial biopsies were obtained by needle arthroscopy at baseline and after 8 weeks of treatment. Clinical disease activity was assessed at baseline and after 8 weeks using the 28-joint Disease Activity Score (DAS28), which incorporates tender and swollen joint counts, patient global assessment, and C-reactive protein (CRP) levels. Treatment failure was defined as a DAS28 improvement <0.6 or a DAS28 >3.7 at week 8. Radiographs of hands and feet were obtained at baseline and one year after treatment initiation to assess typical RA erosions (**Table 1**).

**Table 1:** Baseline characteristics and clinical and radiographic outcomes after adalimumab therapy.

Patient characteristics	RA (N=12)
Sex	Women: 7; men: 5
Age (years)	59.5 (55.0–75.5)
Rheumatoid factor	33.3%
Anti-CCP	Pos: 33.3%; Neg: 33.3%; MD: 33.3%
Disease duration (years)	5 (2–12)
Symptom duration (years)	6 (3.2–13.8)
Good EULAR response	83.3%
ACR20, ACR50, and ACR70 response rate	58.3%; 50.0%; 33.0%
Glucocorticoid use	41.6%
NSAID use	25.0%
DMARD therapy	75.0%
Adalimumab therapy	100%
Erosions at baseline	41.6%
Erosions at 12-month follow-up	41.6%
DAS28-ESR before adalimumab therapy	5.4 (4.3–6.1)
DAS28-ESR after adalimumab therapy	3.1 (2.1–5.2)

Values are presented as the median (interquartile range) or percentages based on the total number of the patients included in the study (n = 12). *Pos* indicates positive, *Neg* indicates negative, and *MD* indicates missing data. Anti-CCP: Anti-cyclic Citrullinated Peptide; ACR: American College of Rheumatology; EULAR: European Alliance of Associations for Rheumatology**;** DMARD: Disease-Modifying Anti-Rheumatic Drug; DAS28: 28-joint Diseases Activity Score; ESR: Erythrocyte Sedimentation Rate**;** NSAID: Non-Steroidal Anti-Inflammatory Drug.

The study (Protocol number 2009/3:7 (2009-08-12), reference number: 2009/865-31/3) was approved by the Ethics Review Board at Karolinska University Hospital, and all participants provided written informed consent.

### 2.2 Immunohistochemistry

Serial sections from synovial biopsies (n = 12) were fixed in 2% formaldehyde and stored at −80 °C. Sections were stained for RANKL, RANK, and OPG using the following primary anti-human monoclonal antibodies: anti-RANKL (12A668; Abcam; 5 µg/mL); anti-RANK (9A725; Abcam; 5 µg/mL); and anti-OPG (MAB805; R&D Systems; 2.5 µg/mL). Cell phenotypes were further characterized using anti-CD3 (347340; T-cell marker; BD Biosciences; 0.04 µg/mL) and anti-CD163 (macrophage marker; DakoCytomation; 0.2 µg/mL). All primary antibodies used in the staining were IgG1, and a concentration-matched mouse IgG1 was used as the negative control (DakoCytomation). Staining was developed using 3,3′-diaminobenzidine (DAB).

Synovial biopsy sections were evaluated semi-quantitatively by three independent observers (AK; SR; AC) blinded to patient identity, biopsy sequence, and treatment response using a four-point scale: 0: no staining/no positive cells; 1, low staining/occasional positive cells; 2, moderate staining/scattered positive cells; and 3, high amount staining/extensive positive cells (15, 29). After independent scoring, inter-observer agreement was assessed using Spearman correlation analysis (r ≥ 0.75; p ≤ 0.035) for most markers. A consensus review was then conducted to reconcile any discrepancies and finalize the scores for analysis.

### 2.3 Cell culture and western blotting

Human SaOS-2 cells were seeded in 10 cm Petri dishes (Corning) at a density of 1×10^6^ cells (DSMZ, Braunschweig, Germany) in McCoy’s 5A medium (Gibco) supplemented with 10% heat-inactivated fetal bovine serum, 2 mM L-glutamine, and antibiotics under standard conditions (37 °C, 5% CO_2_). For experiments, cells were incubated for 24 hours under serum-free conditions with TNF (1 nM = 17.5 ng/mL) (210-TA, R&D systems Minneapolis, USA) in the presence or absence of adalimumab at varying concentrations.

Whole-cell lysates were prepared with RIPA buffer containing 50 mM Tris HCl pH 7.5, 150 mM NaCl, 1% Igepal (Sigma, Mannheim, Germany), 0.5% sodium deoxycholate (Sigma, Mannheim, Germany), and protease inhibitors (Roche Diagnostics GmbH, Mannheim, Germany), and centrifuged at 14,000 rpm for 15 minutes at 4 °C. Protein concentrations were determined using the DC protein assay (Bio-Rad). Equal amounts of protein (40 µg) were separated by SDS-PAGE under non-reducing conditions and transferred to PVDF membranes (Amersham Biosciences, Buckinghamshire, UK). Membranes were blocked with 5% non-fat milk in PBS/Tween-20 and incubated overnight at 4 °C with anti-human RANKL (2 µg/mL) (Abcam, Cambridge, UK), anti-human OPG (1.5 µg/mL) (R&D systems Minneapolis, USA), and rabbit anti-human-β-actin antibodies (Amersham Biosciences, Buckinghamshire, UK) in 0,1% PBS/Tween-20 at 4°C overnight. After washing membrane was incubated with secondary peroxidase linked anti-mouse IgG or peroxidase linked anti-rabbit IgG (Amersham Biosciences, Buckinghamshire, UK) for 1 hour at room temperature. Membrane was washed and protein bands were visualized on X-ray film using enhanced chemiluminescence (Amersham Biosciences, Buckinghamshire, UK). Radiographs were analyzed using Image lab software (Biorad), quantified bands were normalized and corrected with β-actin and results were expressed as arbitrary units.

### 2.4 Osteoclast differentiation and bone resorption

CD14^+^ monocytes were isolated from peripheral blood mononuclear cells (PBMCs) obtained from healthy donor buffy coats using magnetically labelled CD14 microbeads (130-050-201; Miltenyi Biotec, Germany). Cells were cultured in DMEM supplemented with 10% heat-inactivated FBS, 2 mM L-glutamine, 100 IU/mL penicillin, and 50 µg/mL streptomycin (Gibco). CD14^+^ monocytes were seeded at a density of 1x10^5^ cell per well in 96-well plates in triplicates and cultured with human M-CSF (25 ng/mL; 216-MC, R&D Systems) for 3 days. Thereafter, the medium was replenished every 3 days with human RANKL (2 or 50 ng/mL; 390-TN-010) and M-CSF (25 ng/mL), with or without adalimumab (1, 10, or 100 µg/mL) or human IgG1 (control for D2E7).

After 8 days, osteoclasts were stained using a tartrate-resistant acid phosphatase (TRAP) staining kit (387-A; Sigma-Aldrich) according to manufacturer’s instructions. Multinucleated TRAP positive osteoclasts (≥3 nuclei) were counted from a minimum of three replicate wells per treatment using an inverted light microscope. For bone resorption assays, cells were cultured under identical conditions on synthetic calcium phosphate-coated plates (Corning, NY, USA) in 96-well format. After 14 days, cells were removed using 6% chlorine bleach, and resorption area were visualized using a camera connected to the inverted light microscopy. The total resorbed area per well was quantified from a minimum of three replicate wells per treatment by applying live thresholding to the resorbed surface using NIS-Elements software (Nikon Instruments Europe BV, Amsterdam, The Netherlands).

### 2.5 Bio-Plex cytokine assay

Cytokine concentrations in cell-culture supernatants from TNF-α–pre-stimulated Saos-2 cells and adalimumab-treated cultures and osteoclasts cultures were quantified using a bead-based multiplex immunoassay (Bio-Plex Pro™ human cytokine array(10 plex (IL-1β, IL-2, IL-4, IL-10, IL-12(p70), IL-15, IL-17, GM-CSF, IFN-γ and TNF-α and 2 plex IL-6 and IL-8); Bio Rad) according to the manufacturer’s instructions.

### 2.6 Statistical analysis

Comparisons between paired samples were analyzed using the Wilcoxon signed-rank test, whereas comparisons between unpaired independent groups were performed using the Mann–Whitney test. Comparisons involving three or more groups were conducted using ordinary one-way analysis of variance (ANOVA) followed by Tukey’s multiple comparisons test post-hoc test. Differences with p values <0.05 were considered statistically significant. All statistical analyses were performed using GraphPad Prism (version 10.6).

## 3 Results

### 3.1 Adalimumab therapy results in good clinical response with no radiologic progression

Adalimumab treatment demonstrated substantial clinical effectiveness in our setting. Median DAS28 improved from 5.4 (IQR: 4.3–6.1) at baseline (high disease activity) to 3.1 (IQR: 2.1–5.2) after treatment (low disease activity). Among the 12 patients included, 7 achieved an ACR20 response, 6 patients achieved ACR50 response, and 4 patients achieved ACR70 response (30). In addition, 10 patients fulfilled the criteria for good EULAR response 8 weeks after therapy (31). Only one patient showed radiologic progression at the 1-year X-ray follow-up, despite the presence of baseline erosions in 5 of 12 patients (**Table 1**).

### 3.2 Adalimumab reduces synovial inflammation by decreasing synovial macrophage infiltration

**Figure 1:**
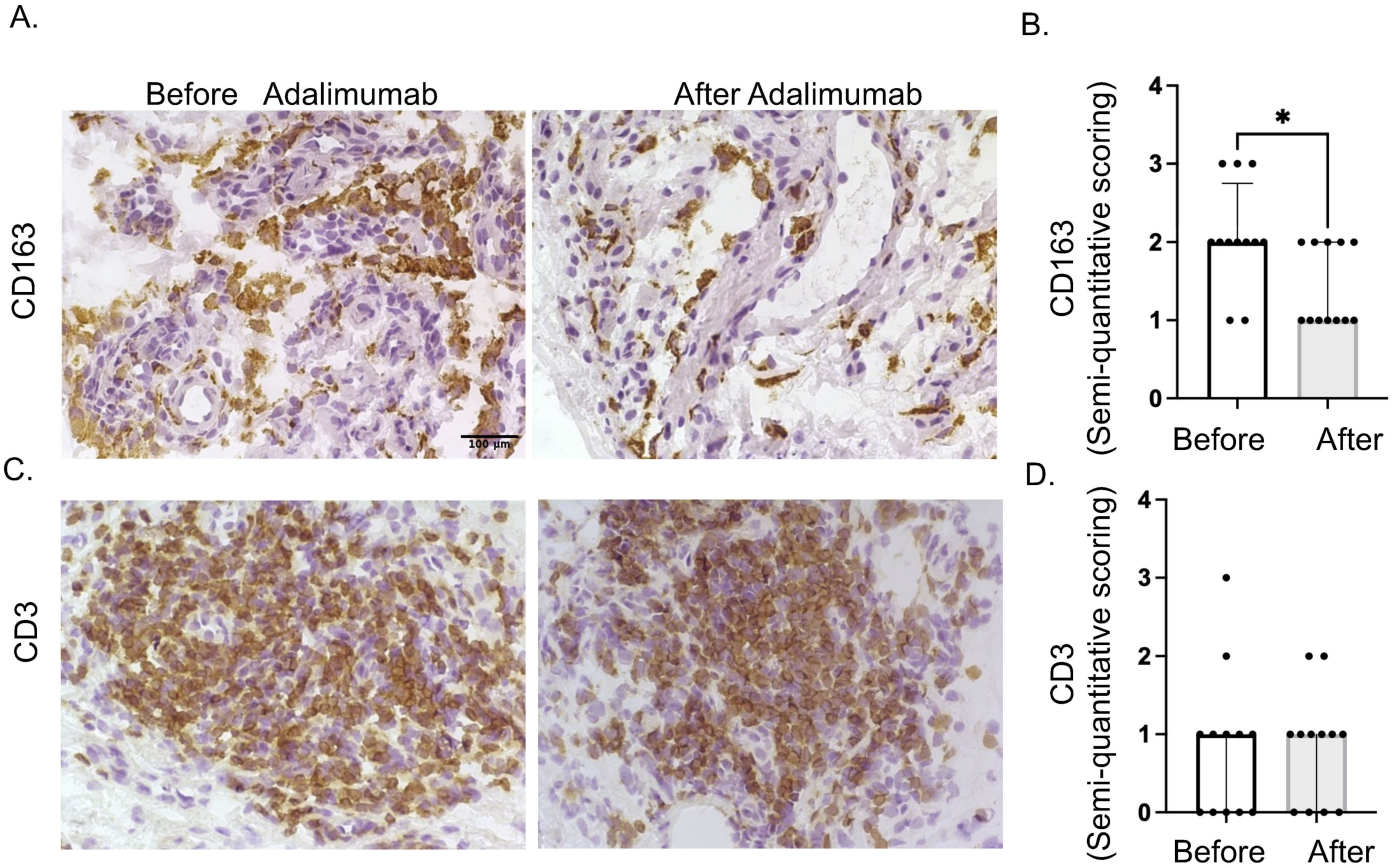
Adalimumab reduces synovial macrophages without altering T−lymphocyte numbers in rheumatoid arthritis. CD163^+^ macrophages and CD3^+^ T lymphocytes were assessed by DAB immunohistochemistry in synovial biopsy specimens obtained before and after treatment with adalimumab. Representative images (250× magnification) show staining for **(A)** CD163^+^ macrophages and **(C)** CD3^+^ T lymphocytes in paired pre− and post−treatment samples. Positively stained cells appear brown, whereas nuclei are counterstained blue with HTX. Semi−quantitative scoring of CD163 and CD3 staining is shown in **(B, D)**, respectively. Data are represented as median semi−quantitative scores. *p < 0.05. CD, cluster of differentiation; DAB, diaminobenzidine; HTX, haematoxylin.

A significant reduction in CD163^+^ macrophage infiltration was observed in the synovial tissue of patients at 8 weeks after adalimumab treatment (from a median of 2 to a median of 1; p = 0.0352), whereas CD3^+^ T-cell numbers remained unchanged (from a median of 1 to a median of 1; p = 0.999), (**Figures 1B, D**). Additional representative paired images illustrating changes in CD163^+^ macrophages and no changes in CD3^+^ T-cells are shown in **Supplementary Figure 1**). A non−significant trend toward a reduction in CD68^+^ macrophages was noted, whereas CD55^+^ fibroblast numbers remained unchanged (**Supplementary Figure 2**) (32).

### 3.3 Adalimumab modulates bone metabolism by reducing the RANKL/OPG ratio *in vivo* and *in vitro*

**Figure 2:**
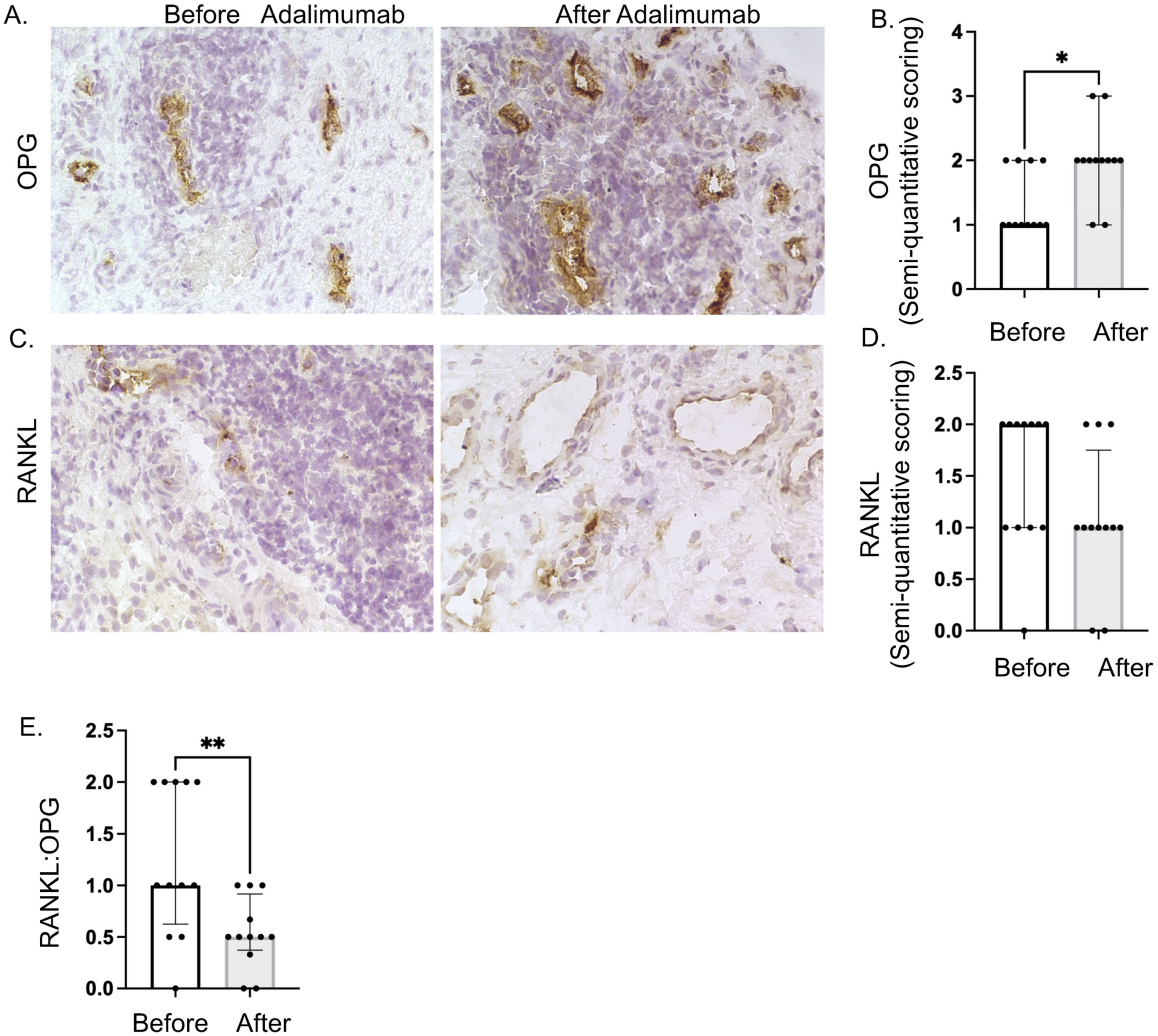
Adalimumab increases synovial OPG expression and reduces the RANKL: OPG ratio in rheumatoid arthritis. OPG and RANKL expression was evaluated by DAB immunohistochemistry in synovial biopsy specimens obtained before and after treatment with adalimumab. Representative images (250× magnification) show staining for **(A)** OPG and **(C)** RANKL in paired pre− and post−treatment samples. Semi−quantitative scoring was used to determine OPG and RANKL expression **(B, D)**, and the RANKL/OPG ratio before and after treatment is shown in **(E)**. Positively stained cells appear brown, whereas nuclei are counterstained blue with HTX. Data are presented as median semi−quantitative scores. *p < 0.05. DAB, diaminobenzidine; HTX, haematoxylin; OPG, osteoprotegrin; RANKL, receptor activator of nuclear factor κB ligand.

Next, we examined expression of the bone-markers RANKL, RANK, and OPG in synovial tissue. OPG levels increased significantly 8 weeks after adalimumab treatment (from a median of 1 to a median of 2; p = 0.0312), (**Figures 2A, B**). RANKL levels decreased but did not change significantly at the group level (from a median of 2 to a median of 1; p = 0.12) (**Figures 2C, D**). We noted a significant reduction in the RANKL: OPG ratio, indicating a shift towards a less osteoclastogenic milieu (from a median of 1 to a median of 0.5; p = 0.0049), (**Figure 2E**), while no effect was observed in RANK expression (**Supplementary Figure 2**).

**Figure 3:**
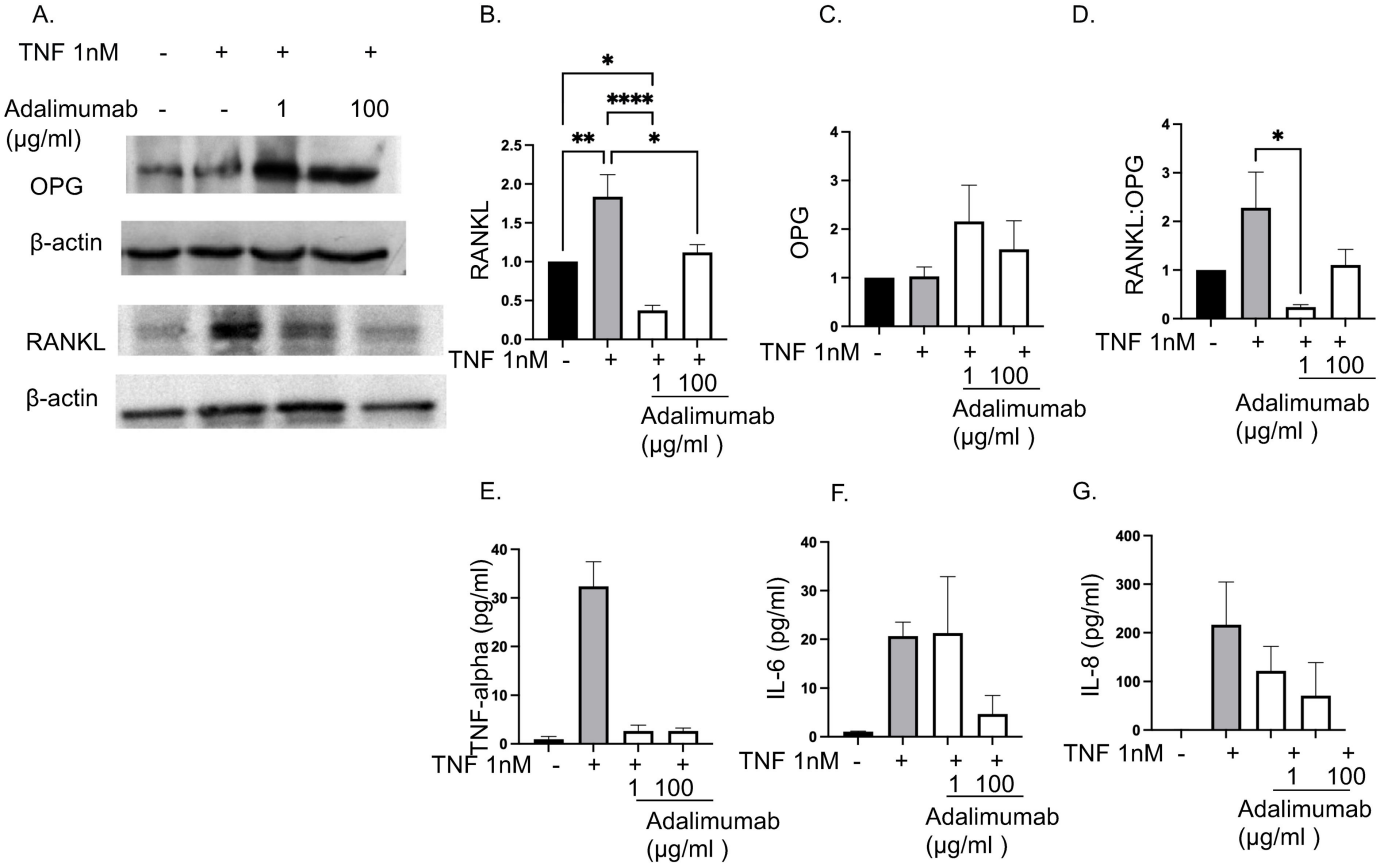
Adalimumab increases OPG expression and decreases RANKL expression in TNF-primed osteoblast-like SaOS-2 cells. SaOS-2 cells were pre-stimulated with TNF- α, washed, and then treated with adalimumab for 24 hours. Receptor activator of nuclear factor κB ligand RANKL and OPG expression were analyzed by Western blotting. **(A)** Representative blots showing RANKL and OPG expression with β-actin as a loading control. **(B, C)** RANKL and OPG band intensities were quantified by densitometry and normalised to β-actin, then expressed relative to the non-TNF-treated control. **(D, E)** RANKL/OPG ratio in adalimumab-treated cultures, based on densitometric measurements from five independent experiments. Cell−culture supernatants were collected from TNF-alpha primed SaOS-2 after 24 hours of adalimumab treatment, and cytokine concentrations were quantified using a Bio−Plex multiplex cytokine assay. **(E–G)** Concentrations of TNF-α, IL-6, and IL-8, and in the SaOS-2 culture supernatants are shown from two independent experiments. Data are presented as the mean ± SEM. *p < 0.05. IL, interleukin; OPG, osteoprotegrin; RANKL, receptor activator of nuclear factor κB ligand; SaOS2, Sarcoma Osteogenic-2; SEM, standard error of the mean; TNF- α, tumor necrosis factor alpha.

To better understand the changes in the RANKL, RANK, and OPG axis, SaOS-2 cells were chosen, with the premise that they have similar properties to those of human osteoblasts (33, 34). SaOS-2 cells were pre-stimulated with TNF for 24 hours, washed and subsequently treated with adalimumab at 1 and 100 μg/mL. Western blot analysis demonstrated a significant decrease in the TNF induced RANKL expression and a concomitant increase in OPG levels following adalimumab treatment (**Figures 3A–D**). Notably, the RANKL: OPG ratio was significantly reduced by adalimumab 1 μg/mL, from a median of 1.38 (IQR 1.00–4.70) to 0.25 (IQR 0.14–0.38); p = 0.011 (**Figure 3D**).

We also observed that TNF blockade decreased TNF-induced pro-inflammatory cytokines (TNF- α, IL-6, and IL-8) in SaOS-2 culture supernatants (**Figures 3E–G**), suggesting a broader cytokine network effect whereby TNF-α inhibition with adalimumab dampens multiple inflammatory mediators.

To evaluate the osteoclast stimulatory potential of the SaOS-2 cells, we cultured M-CSF-stimulated CD14^+^ macrophages with supernatants from SaOS-2 cells cultured with and without TNF−α. Compared to a non-stimulated control, TNF−α stimulation resulted in a significant increase in TRAP−positive osteoclast precursors (mean ± SEM: 82 ± 18 to 295 ± 59; p = 0.0043) and a trend toward increased multinucleated osteoclasts (10 ± 1.7 to 14 ± 0.83; p = 0.1; **Supplementary Figure 3**).

### 3.4 Adalimumab directly inhibits RANKL-mediated osteoclastogenesis and bone erosion

**Figure 4:**
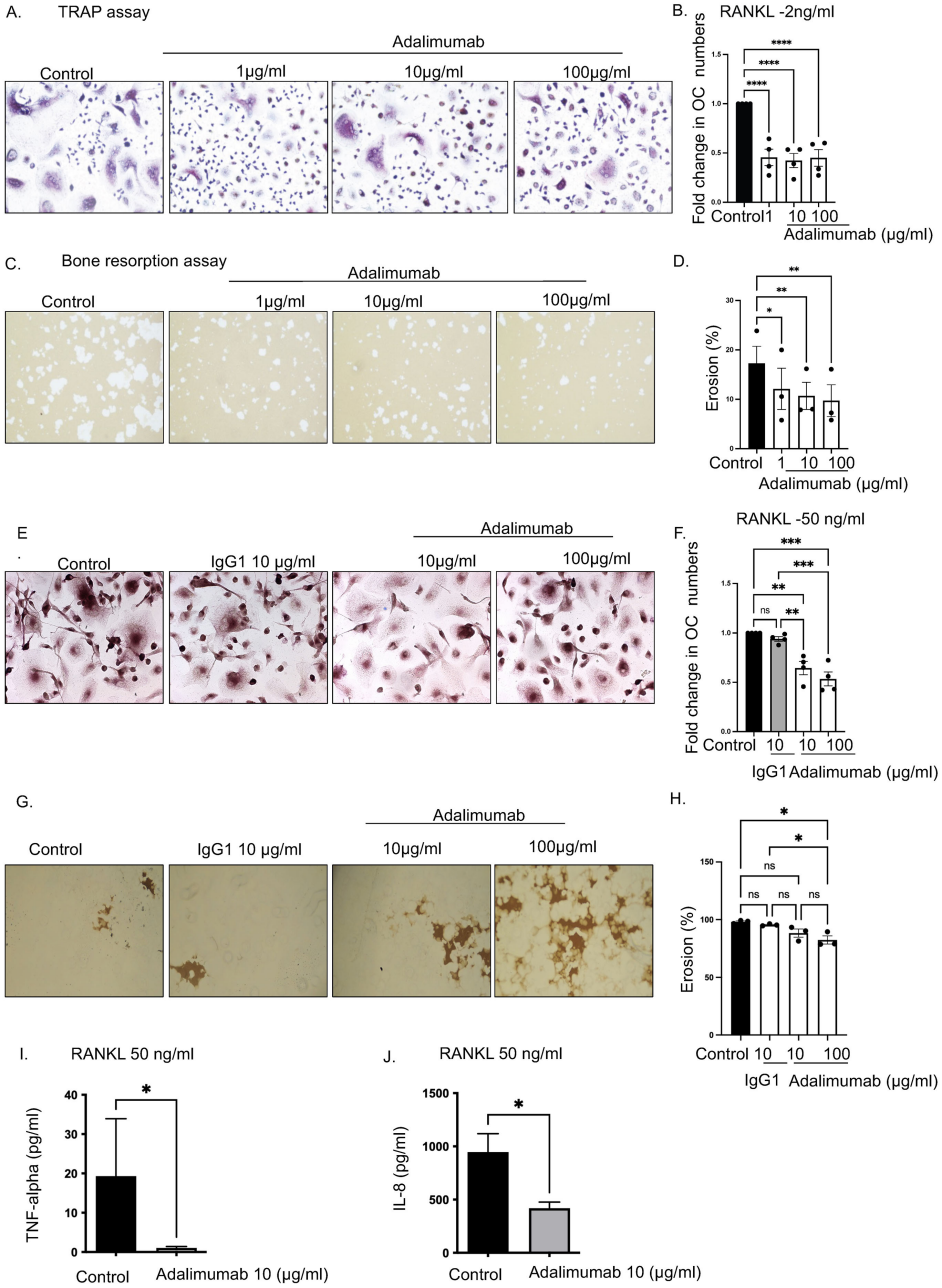
Adalimumab inhibits osteoclastogenesis and bone resorption *in vitro*. **(A, E)** Representative TRAP-stained images of osteoclasts generated from peripheral blood CD14^+^ monocytes from healthy donors. Cultures were treated with human IgG1 (control) or adalimumab (1, 10, and 100 µg/mL) in the presence of M-CSF and low RANKL (2 and 50 ng/mL). TRAP-positive multinucleated cells (≥3 nuclei) were counted as osteoclasts. **(B, F)** Quantification of osteoclast numbers expressed as fold change relative to RANKL stimulation (2 or 50 ng/mL), based on at least four independent experiments. **(C, G)** Bone resorption assay performed on synthetic bone surfaces. Representative images show resorption pits (white) against intact bone surface (brown) from low RANKL (2ng/ml). **(D, H)** Quantification of erosion area across treatments using image analysis software and expressed as the percentage of total surface eroded, based on at least three independent experiments with RANKL stimulation (2 or 50 ng/mL). **(I, J)** Adalimumab treatment significantly reduced TNF-α and IL-8 production in M-CSF/RANKL-stimulated macrophages, as determined by Bio-Plex multiplex cytokine assay readouts from at least three independent donors. Data are presented as the mean ± SEM. *p < 0.05. CD, cluster of differentiation; M-CSF, macrophage colony stimulating factor; RANKL, receptor activator of nuclear factor κB ligand; SEM, standard error of the mean; TRAP, tartrate resistant acid phosphatase.

To assess the impact of adalimumab on osteoclastogenesis, CD14^+^ monocytes from healthy donors were stimulated with M-CSF for macrophage generation and then differentiated into osteoclasts in the presence of increasing concentrations of adalimumab. After 8 days of culture, TRAP positive osteoclast numbers were quantified. Adalimumab significantly reduced osteoclast numbers under low RANKL conditions (2 ng/mL; **Figures 4A, B**). The inhibitory effect was comparable across all tested adalimumab concentrations (1, 10, and 100 μg/mL), with significant reductions compared to control (mean ± SEM: 0.45 ± 0.08, 0.45 ± 0.08, and 0.42 ± 0.07, respectively).

Osteoclast function was next evaluated using a synthetic bone resorption assay. At RANKL concentrations 2 ng/mL, approximating those reported in the serum of patients with RA, adalimumab-treated cultures displayed a marked reduction in erosion area at all tested doses (mean ± SEM: 17.30 ± 3.4% in control compared to 9.7 ± 3.4%, 12.1 ± 4.1%, and 10.6 ± 2.7% with adalimumab at 1, 10, and 100 µg/ml, respectively; **Figures 4C, D**). Under high RANKL conditions (50 ng/mL), adalimumab significantly reduced osteoclast numbers in a dose-dependent manner (mean ± SEM: 0.64 ± 0.06-fold (10 μg/mL) and 0.53 ± 0.07-fold (100 μg/mL) compared to control). In contrast, the IgG1 control antibody had no effect on osteoclast formation (**Figures 4E, F**). Despite this decrease in osteoclast numbers, a significant reduction in bone erosion was observed only at the highest adalimumab concentration (98 ± 0.8% in control and 95.4 ± 0.4% in IgG1 10 μg/mL control compared to 88.3 ± 3.5% and 82.3 ± 3.5% with adalimumab 10 μg/mL and 100 μg/mL treatment, respectively), with no detectable effect at lower doses (**Figures 4G, H**). To further assess the effect of adalimumab on pro-inflammatory cytokine production during osteoclast differentiation, we quantified multiple cytokines in day 7 high RANKL osteoclast culture supernatants from the bone erosion assay using a Bio-Plex assay. TNF-α and IL-8 levels were significantly reduced following the addition of adalimumab (**Figures 4I, J**).

Collectively, these findings indicate that adalimumab suppresses osteoclast differentiation and attenuates bone resorption *in vitro*, supporting a direct inhibitory effect on osteoclast-mediated bone destruction.

## 4 Discussion

In this study, we provide translational evidence on how TNF-α blockade with adalimumab contributes to bone protection in rheumatoid arthritis by integrating clinical outcomes, analysis of 12 paired synovial biopsies, and mechanistic *in vitro* experiments. Using a well-characterized clinical cohort, we observed that most patients treated with adalimumab in combination with a synthetic DMARD achieved a good clinical response without radiographic progression over one year, despite baseline erosions in several cases. These findings are consistent with previous large-scale studies showing that TNF-α inhibition mitigates structural damage and preserves bone integrity in RA (24, 26).

Previously, we reported increased TNF-α expression in synovial tissue and observed reduced synovial TNF-α levels in responders after TNF-α blockade (29). In the current analysis, synovial tissue examination revealed a significant reduction in CD163^+^ macrophages following adalimumab treatment. CD163^+^ macrophages are conventionally considered to shape an M2-like anti-inflammatory phenotype; however, their role in rheumatoid synovial tissue may be more complex and context-dependent. Recent single-cell sequencing has identified CD163^+^CD206^+^ macrophages to correlate with disease activity and they may play a pathogenic role (16, 23). CD163^+^ macrophage reduction suggests that TNF blockade may promote efflux or apoptosis of pathogenic macrophage subsets, thereby reducing local inflammation (35). In addition, circulating soluble CD163, which is released by the action of TACE (TNF-α converting enzyme), has been proposed as a marker of radiographic progression in early RA (18, 36). We observed heterogeneity in T-cell distribution patterns, with germinal center-like aggregates present in some patients, while most exhibited a diffuse cellular pattern (37). Despite this variability, CD3+ T-cell numbers remained unchanged following adalimumab treatment, indicating that adalimumab may not directly modulate local synovial T-cell infiltration (37). However, this does not preclude systemic immunomodulatory effects, as adalimumab has been reported to increase the frequency and suppressive function of regulatory T-cells in peripheral blood (38).

We further demonstrated that adalimumab modulates the RANKL/OPG axis both in RA synovium and *in vitro*. In synovial tissue, OPG expression was prominently increased in the vascular endothelial cells after adalimumab treatment, and the reduced RANKL/OPG ratio indicate a shift towards a less osteoclastogenic environment, providing a plausible mechanism for bone protection. While we did not observe a significant decrease in RANKL levels in the synovial tissue, *in vitro* experiments using TNF-α-primed SaOS-2 cells showed that anti-TNF-α treatment with adalimumab significantly decreased RANKL and with marginal increase in OPG expression. In addition to our current observation of increased OPG in vasculature in synovial tissue, a decrease in the RANKL/OPG ratio in human dermal microvascular endothelial cells (HDMECs) has been reported previously. This modulation likely occurs through neutralization of TNF-α, which binds TNFR-I and TNFR-II, signaling via TRAF2-mediated NF-κB activation (9, 15, 39, 40). TNF-α blockade also reduced IL-6 and IL-8 levels, cytokines capable of promoting RANKL-independent osteoclastogenic pathways (4, 41). Additionally, we demonstrated the osteoclastogenic potential of SaOS-2 cell-conditioned medium is enhanced by TNF-α. While previous studies using infliximab primarily reported decreases in the RANKL: OPG ratio (15), our findings further indicate that pro−inflammatory soluble mediators may also contribute to this process, suggesting that adalimumab exerts broader immunoregulatory effects on the repertoire of released pro−osteoclastogenic factors.

CD14^+^ monocytes stimulated with M-CSF can acquire a CD163^+^/CD68^+^ phenotype resembling synovial tissue resident macrophages (3, 16). In our study, macrophages with similar characteristics secreted soluble TNF-α in response to RANKL and M-CSF, consistent with their ability to express membrane-bound TNF-α and shed its soluble form (42). Although TNF-α alone does not induce human osteoclastogenesis, it can markedly potentiate RANKL-mediated osteoclast differentiation (43–45). This mechanism is particularly relevant in the context of ACPA-positive RA, in light of our prior work showing that osteoclast-specific ACPAs (such as the monoclonal ACPA 1325:C03 human IgG1) and patient-derived polyclonal ACPAs induce osteoclastogenesis through an IL-8-dependent pathway, with autocrine IL-8 being the key signal required for osteoclast maturation (3, 4, 46). In the present study, adalimumab inhibited osteoclastogenesis and bone resorption under both low and high RANKL conditions. This may reflect neutralization of soluble and membrane-bound TNF-α, reducing TNF-dependent amplification of RANKL signaling (47). Notably, we observed TNF-α production under high RANKL conditions but not at low RANKL concentrations, whereas IL-8 was detectable under both conditions. Importantly, adalimumab treatment also indirectly decreased IL-8 levels in both osteoclast and osteoblast supernatants, suggesting downregulation of the autocrine IL-8 loop that promotes osteoclast maturation, a mechanism that may be particularly important in ACPA-positive patients where this pathway is upregulated (3, 4).

A key mechanistic link resides in the differential response of monocyte subsets to TNF stimulation, which predominantly upregulates TNFR2 expression on classical monocytes (48). This receptor expression pattern confers heightened sensitivity of osteoclast precursors to TNF signaling, thereby promoting their differentiation into bone-resorbing osteoclasts. Our findings support this mechanistic framework by demonstrating that adalimumab treatment significantly reduced the CD163^+^ macrophage population in the synovial tissue of patients with RA, suggesting effective targeting of the monocyte-macrophage-osteoclast axis.

The bone-protective effects of TNF-α blockade extend beyond RA and are well-established in other inflammatory arthropathies, including psoriatic arthritis and ankylosing spondylitis, where bone erosion and systemic bone loss represent cardinal disease features (49–51). This cross-disease efficacy suggests that adalimumab-mediated inhibition of monocyte-to-osteoclast differentiation represents a conserved therapeutic mechanism across the spectrum of inflammatory arthropathies. Nevertheless, disease-specific immunological factors, such as ACPA-driven bone loss in RA or the distinct IL-23/IL-17 axis predominance in axial spondyloarthritis, likely modulate the magnitude and kinetics of this therapeutic response.

**Figure 5:**
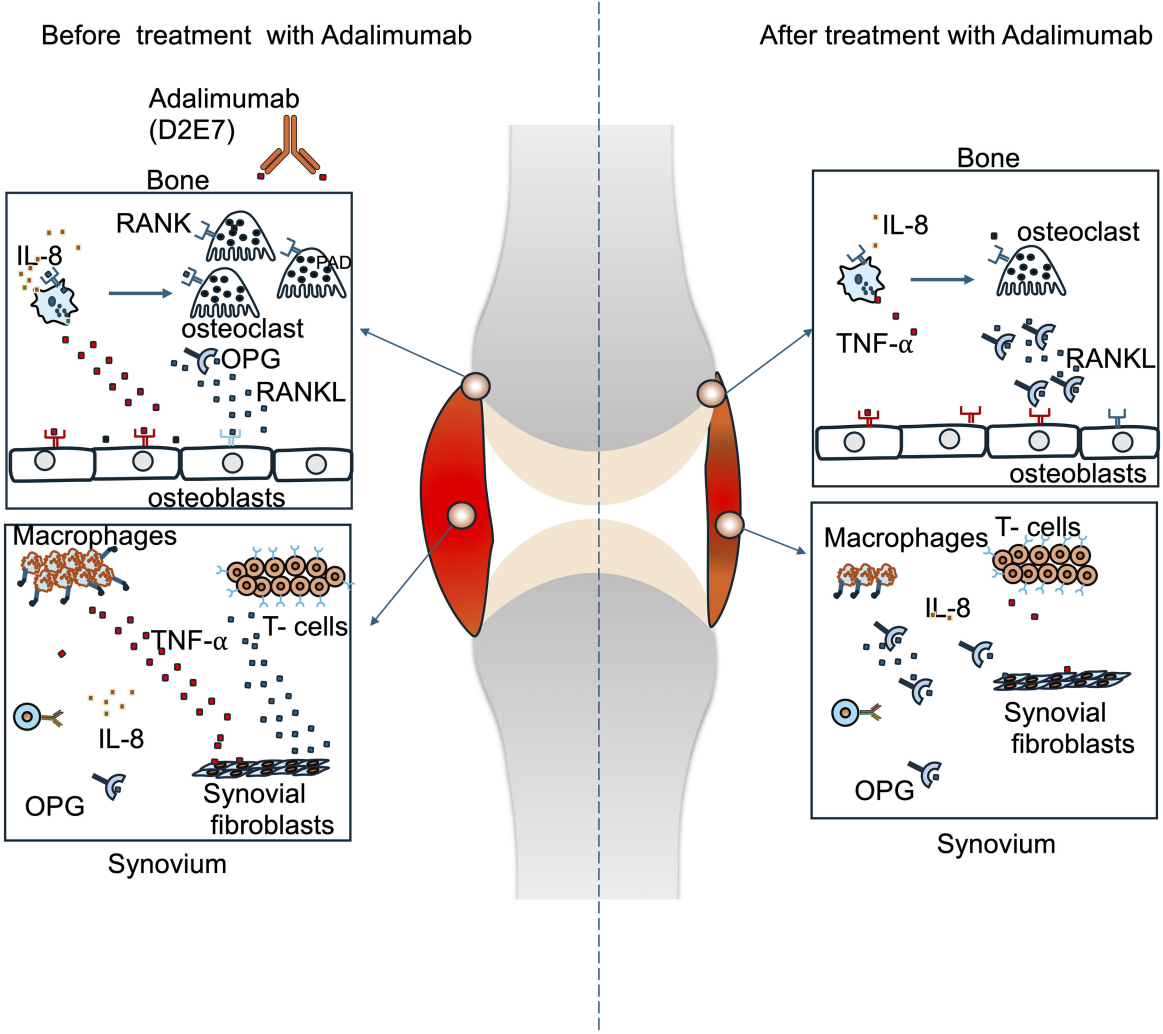
Cellular and cytokine changes underlying the bone−protective effects of adalimumab. Adalimumab could modify circulating monocytes and resident synovial macrophages without altering synovial T cells or fibroblasts. TNF−α neutralization promotes macrophage efflux from the synovium, reduces IL−6 and IL−8 production, and shifts local bone−regulatory mediators by decreasing RANKL and increasing OPG expression. In osteoblasts, TNF−α inhibition similarly restores the RANKL/OPG balance. In osteoclasts, TNF−α inhibition directly inhibits the macrophage-derived osteoclastogenesis and bone erosion. Together, these coordinated cellular and cytokine changes contribute to improved bone metabolism and attenuation of synovial inflammation.

Taken together, our findings suggest that adalimumab confers bone-protective effects through complementary mechanisms: reducing synovial macrophage infiltration, restoring a protective RANKL/OPG balance, and directly suppressing osteoclast differentiation and bone resorption. Adalimumab also influences osteoblast by shifting the RANKL/OPG ratio, thereby indirectly limiting the maturation of osteoclast precursors (**Figure 5**).

Several limitations should be acknowledged. Although we analyzed 12 paired synovial biopsies before and after adalimumab treatment, most patients received concomitant synthetic DMARDs, which may enhance therapeutic responses in RA (52). Our *in vitro* assays did not include these co-treatments and therefore reflect the direct cellular effects of adalimumab under controlled conditions.

Overall, our findings highlight TNF-α as a central driver of inflammatory bone erosion in RA and show that its blockade reverses key pathways promoting osteoclastogenesis. The combined synovial and *in vitro* findings provide a coherent mechanistic basis for the bone-sparing properties of adalimumab and support early TNF-α inhibition to prevent structural damage alongside controlling synovial inflammation.

## Data Availability

The raw data supporting the conclusions of this article will be made available by the authors, without undue reservation.
