# Supplementary material for: TNF-α blockade with adalimumab restores bone metabolism in rheumatoid arthritis by regulating cytokines and inflammatory cell infiltration
Source: Front Immunol. 2026 Jul 23;17:1866519. doi: 10.3389/fimmu.2026.1866519 (PMC13442820; doi:10.3389/fimmu.2026.1866519)
Supplement: Supplementary file 1 [file DataSheet1.zip › Data Sheet 1/Supplementary Figure S1..docx]

**Supplementary Figure S1. Representative images of histological scoring for paired sections of CD3, CD163, OPG, and RANKL.**

Synovial biopsies obtained from patients before and after adalimumab treatment were sectioned and stained for CD3, CD163, OPG, and RANKL markers. (A) CD3 staining showing a dispersed pattern in synovial tissue, demonstrating CD3^+^ T cell infiltration with no significant change in expression between baseline and 8 weeks after adalimumab treatment. (B) CD163 staining in synovial tissue showing marked reduction in CD163^+^ macrophage infiltration following adalimumab treatment compared to baseline, indicating reduced macrophage-mediated inflammation. (C) OPG and RANKL staining in synovial tissue demonstrating increased OPG expression in the vasculature following adalimumab treatment, while RANKL expression remained unchanged. All images were acquired at 100× magnification. Abbreviations: OPG, osteoprotegerin; RANKL, receptor activator of nuclear factor kappa-B ligand; CD, cluster of differentiation.
