# Supplementary material for: TNF-α blockade with adalimumab restores bone metabolism in rheumatoid arthritis by regulating cytokines and inflammatory cell infiltration
Source: Front Immunol. 2026 Jul 23;17:1866519. doi: 10.3389/fimmu.2026.1866519 (PMC13442820; doi:10.3389/fimmu.2026.1866519)
Supplement: Supplementary file 1 [file DataSheet1.zip › Data Sheet 1/Supplementary Figure S3..docx]

**Supplementary Figure S3. TNF‑α enhances SaOS-2 cells mediated osteoclastogenesis.**

Osteoclast differentiation assays were performed using macrophages cultured with supernatants from SaOS-2 cells treated with or without TNF-α for 24 hours. Osteoclasts were identified and quantified using tartrate-resistant acid phosphatase (TRAP) staining. (A) Representative images showing the TRAP-positive osteoclast precursors and multinucleated mature osteoclasts following TNF-α treatment. (B) Quantification of TRAP-positive mononuclear osteoclast precursor numbers (cells with <3 nuclei). (C) Quantification of TRAP-positive multinucleated osteoclast numbers (cells with ≥3 nuclei). Results represent values from at least two independent donors. Abbreviations: TRAP, tartrate-resistant acid phosphatase; TNF-α, tumor necrosis factor alpha.
