# Supplementary material for: TNF-α blockade with adalimumab restores bone metabolism in rheumatoid arthritis by regulating cytokines and inflammatory cell infiltration
Source: Front Immunol. 2026 Jul 23;17:1866519. doi: 10.3389/fimmu.2026.1866519 (PMC13442820; doi:10.3389/fimmu.2026.1866519)
Supplement: Supplementary file 1 [file DataSheet1.zip › Data Sheet 1/Supplementary Figure S2..docx]

**Supplementary Figure S2. Adalimumab does not significantly alter synovial RANK, CD68, or CD55 expression.** Expression of RANK, CD68⁺ macrophages, and CD55⁺ fibroblasts was evaluated by DAB immunohistochemistry staining in synovial biopsy specimens obtained before and after treatment with adalimumab. Representative images (250× magnification) show staining for (A, B, C) RANK, CD68, and CD55 in paired pre‑ and 8 weeks post‑treatment samples. Semi‑quantitative scoring was used to determine expression of (B) RANK, (D) CD68, and (F) CD55 before and after treatment. Data are presented as the median with interquartile range for semi‑quantitative scores. **p* < 0.05. Abbreviations: CD: cluster of differentiation; DAB: diaminobenzidine; RANK: receptor activator of nuclear factor κB.
